# Tsetse Salivary Gland Hypertrophy Virus: Hope or Hindrance for Tsetse Control?

**DOI:** 10.1371/journal.pntd.0001220

**Published:** 2011-08-30

**Authors:** Adly M. M. Abd-Alla, Andrew G. Parker, Marc J. B. Vreysen, Max Bergoin

**Affiliations:** 1 Insect Pest Control Laboratory, Joint FAO/IAEA Division of Nuclear Techniques in Food and Agriculture, Vienna, Austria; 2 Laboratoire de Pathologie Comparée, Université Montpellier 2, Montpellier, France; International Centre of Insect Physiology and Ecology, Kenya

## Abstract

Many species of tsetse flies (Diptera: Glossinidae) are infected with a virus that causes salivary gland hypertrophy (SGH), and flies with SGH symptoms have a reduced fecundity and fertility. The prevalence of SGH in wild tsetse populations is usually very low (0.2%–5%), but higher prevalence rates (15.2%) have been observed occasionally. The successful eradication of a *Glossina austeni* population from Unguja Island (Zanzibar) using an area-wide integrated pest management approach with a sterile insect technique (SIT) component (1994–1997) encouraged several African countries, including Ethiopia, to incorporate the SIT in their national tsetse control programs. A large facility to produce tsetse flies for SIT application in Ethiopia was inaugurated in 2007. To support this project, a *Glossina pallidipes* colony originating from Ethiopia was successfully established in 1996, but later up to 85% of adult flies displayed symptoms of SGH. As a result, the colony declined and became extinct by 2002. The difficulties experienced with the rearing of *G. pallidipes*, epitomized by the collapse of the *G. pallidipes* colony originating from Ethiopia, prompted the urgent need to develop management strategies for the salivary gland hypertrophy virus (SGHV) for this species. As a first step to identify suitable management strategies, the virus isolated from *G. pallidipes* (GpSGHV) was recently sequenced and research was initiated on virus transmission and pathology. Different approaches to prevent virus replication and its horizontal transmission during blood feeding have been proposed. These include the use of antiviral drugs such as acyclovir and valacyclovir added to the blood for feeding or the use of antibodies against SGHV virion proteins. In addition, preliminary attempts to silence the expression of an essential viral protein using RNA interference will be discussed.

## Introduction

Tsetse flies (*Glossina* spp.) are the only cyclical vectors of two debilitating diseases in Africa, sleeping sickness in humans (human African trypanosomosis [HAT] caused by *Trypanosoma brucei gambiense* and *Trypanosoma brucei rhodesiense*) and the cattle disease nagana (African animal trypanosomosis [AAT] caused by *T. b. brucei*, *Trypanosoma congolense*, and *Trypanosoma vivax*) [1], [2]. Nagana, and in certain areas also sleeping sickness, have been a major obstacle to sub-Saharan African rural development and a severe constraint to agricultural production [3]. Due to the lack of effective vaccines and inexpensive drugs for HAT, and the development of resistance of the AAT parasites against available trypanocidal drugs [4], vector control remains the most efficient strategy for the sustainable management of these diseases [5].

**Figure 1:**
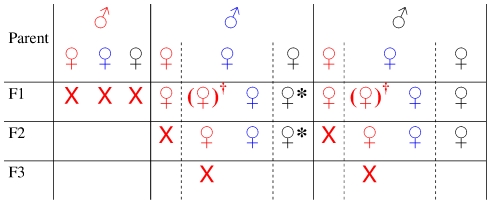
Vertical transmission pattern of the SGHV. Red, hypertrophied; blue, infected but not hypertrophied; black, uninfected. *: Not confirmed, as no virus free colony is available. X: No progeny (sterile). **†**: In each generation, a small proportion of the progeny of infected asymptomatic females develop SGH.

Successful eradication of *Glossina austeni* from the island of Unguja, United Republic of Tanzania, was achieved using an area-wide integrated pest management approach [6] that included the release of sterile male flies [7]. As a consequence of this success, programs were developed to apply this approach on the African mainland and, in 1996, the government of Ethiopia embarked on such a program with the aim of creating a zone free of *Glossina pallidipes* in the Southern Rift Valley of Ethiopia [8], [9]. This project included the establishment of a laboratory colony of the target species at the Insect Pest Control Laboratory (former Entomology Unit) of the Joint FAO/IAEA Programme of Nuclear Techniques in Food and Agriculture, Seibersdorf, Austria. Following its successful establishment using pupae obtained from the target field population in Ethiopia, the colony experienced a steady decline over 2 years and finally became extinct. Investigations revealed that up to 85% of both male and female flies had salivary gland hypertrophy (SGH), a syndrome first described in wild populations of *G. pallidipes* [10], [11], but later detected in many tsetse species from different African countries [12]–[19]. Jaenson [20] was the first to identify a nuclear rod-shaped enveloped DNA virus averaging 70 nm×640 nm in size as the causative agent. This virus was also associated with testicular degeneration and ovarian abnormalities [14], [21]–[23] and affected the development, survival, fertility, and fecundity of naturally [24] or experimentally [25], [26] infected flies. In tsetse field populations, mother-to-offspring transmission, either trans-ovum or through infected milk glands, is thought to be the most likely mode of transmission of the virus (Figure 1) [15], [23], [27]. In laboratory-maintained flies, horizontal transmission during in vitro feeding of blood provided under a silicone membrane [28] was suspected to be a significant route of virus infection, as each tray of blood may be used to feed up to ten successive sets of fly cages. The complete genome of this virus, now designated as the *G. pallidipes* salivary gland hypertrophy virus (GpSGHV), has been sequenced [29]–[32]. In order to better understand the dynamics and mode of transmission of the virus under laboratory rearing conditions, simple and reliable PCR and qPCR methods were developed [33], [34] and studies on the dynamics of the virus in the laboratory colonies were initiated [35].

This paper reviews data on the biology, epidemiology, transmission, and dynamics of the GpSGHV in field populations and laboratory colonies and describes potential strategies to manage the virus' impact in tsetse laboratory colonies. The limitations that hinder the use of this virus as a biological control agent for tsetse control are likewise discussed.

## Methodology

Articles were identified by searching Medline through PubMed using various combinations of terms, including “Salivary gland hypertrophy virus”, “tsetse”, “SIT”, “RNAi”, “Antibodies neutralization”, and “Antiviral drugs”. Research papers and case reports from African countries were retrieved. Additional articles were obtained by citation tracking of review and original articles. The review also drew on conference proceedings and original research conducted by the authors.

## Epidemiology of SGHV in Tsetse Fly Field Populations

Since the first record of SGH in 1934 by Whitnall [36], several observations have given insight into the epidemiology of SGHV: (i) the SGH prevalence in wild tsetse populations was in general low (0.5%) but could reach up to 15% [13], [17], [20], [37], [38], (ii) most of the wild flies with SGH were young flies [39], (iii) some flies with SGH contained blood in the gut, indicating their ability to fly and feed [17], (iv) males with SGH were almost always sterile and females with SGH were only partially fertile [20], and (v) flies with SGH had a shortened life span and often had difficulties feeding that might be caused by a potential reduction of salivary gland secretion and anticoagulant activity [26]. Furthermore, we recently showed that in laboratory colonies (i) flies without SGH could be asymptomatically infected [33], [34], (ii) all the progeny from females with SGH developed SGH, had high virus loads, and were sterile [35], and (iii) in experimental field cage (dimensions: diameter 2.9 m, height 2.0 m) studies, males with SGH showed reduced mating competitivness, but remating frequency of females was very low irrespective of the SGH status of the males in the first mating (unpublished data).

Based on these previous observations, we constructed a model of the vertical transmission of the virus in tsetse populations (Figure 1). In each population, three types of flies (males and/or females) can be present with respect to SGHV infection status: (a) healthy (uninfected) flies, (b) asymptomatic infected flies (low virus load), and (c) symptomatic infected flies (SGH and high virus load). With respect to vertical transmission within these populations, symptomatic males or females produce no progeny or sterile progeny, respectively. The reduced life span of symptomatic flies along with the sterility of their progeny (symptomatic mothers) and the absence of progeny (symptomatic fathers) explain their low prevalence (0.5%) in wild populations. The only way for the virus to propagate through vertical transmission in wild fly populations is by mating of asymptomatic infected flies either amongst each other or with a healthy partner. Since the transmission is either trans-ovum or through the infected milk glands [27], the progeny status will depend, most probably, on the infection status of the mothers. Preliminary PCR analyses of sampled wild tsetse from different African areas revealed a high prevalence of asymptomatic infected flies (unpublished data).

**Figure 2:**
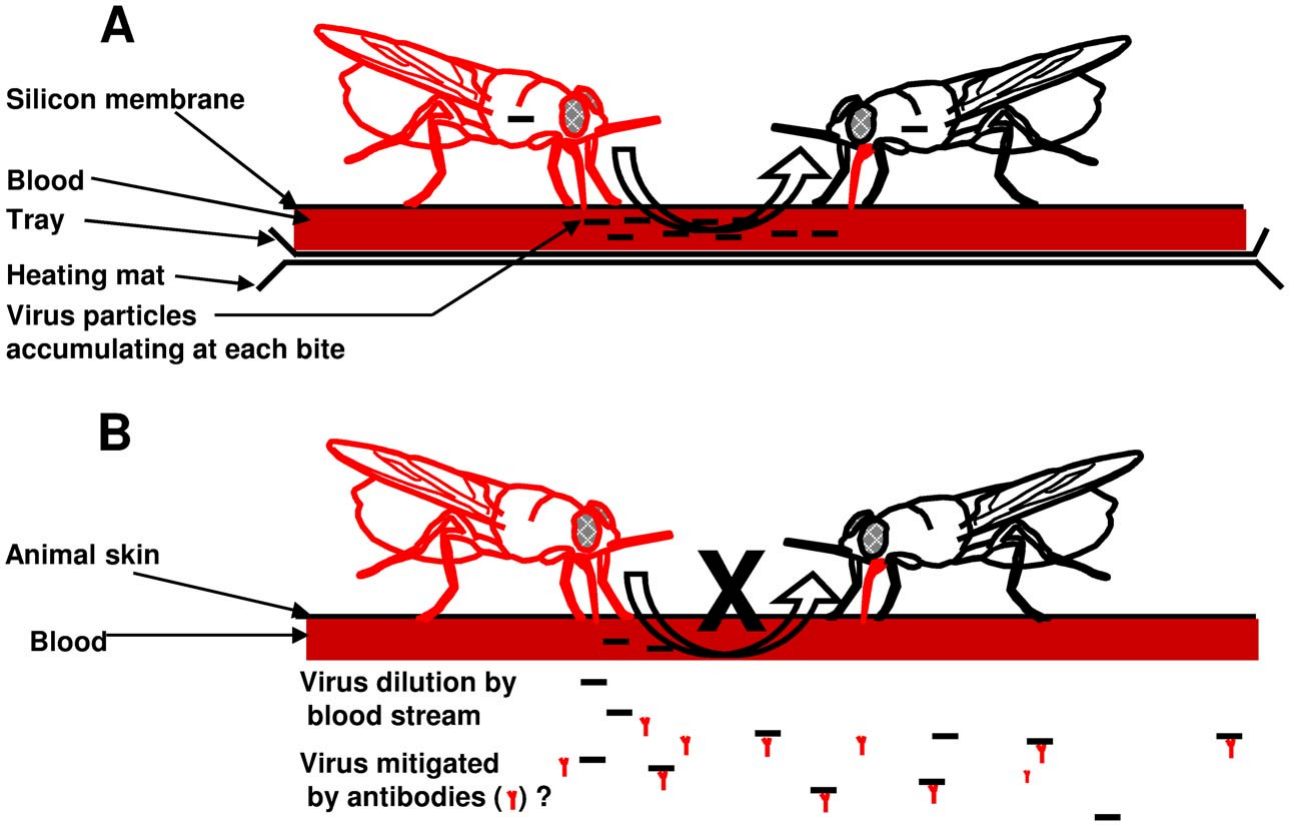
Horizontal transmission of SGHV in *G. pallidipes*. (A) In vitro membrane feeding. (B) In vivo animal feeding. Red, flies with SGH; black, uninfected flies.

We have recently demonstrated that the virus released by infected flies during feeding on an in vitro membrane feeding system is an important source of contamination under laboratory conditions [35] (Figure 2A). Under field conditions, tsetse flies take their blood meals by feeding on wild or domestic animals. The virus inoculum injected into animal by symptomatic or asymptomatic infected flies during blood feeding is probably rapidly diluted in the blood stream, and the probability of healthy flies becoming infected by taking their blood meal on the same animal is consequently very low. Furthermore, we recently demonstrated that rabbits used to feed hundreds of asymptomatic infected flies per week in laboratory conditions developed virus-specific antibodies (unpublished data; Figure 2B). Assuming that the amount of virus inoculated by repeated blood meals of infected flies on the same animal is sufficient to develop such antibodies, the virus particles injected into this animal might be neutralized. So far, no investigations have been carried out in Africa to detect SGHV-specific antibodies in the blood of domestic or wild mammals on which tsetse flies normally feed.

## Epidemiology of SGHV in Tsetse Fly Laboratory Colonies

In contrast to field conditions, the prevalence of SGH in tsetse laboratory colonies can increase up to 85% in certain cases. This high prevalence was responsible for the collapse in 1987 of the *G. pallidipes* colony established in 1983 at the Insect Pest Control Laboratory collected from a population in the Lambwe Valley, Kenya, and in 2002 of a colony established in 1996 from Arba Minch, Ethiopia. The collapse of these colonies prompted research to understand the modalities of virus transmission under laboratory conditions and to develop potential virus management strategies [33]. These investigations have shed light on some crucial points in the virus transmission and dynamics in tsetse colonies. Although vertical transmission from mother to progeny also occurs in laboratory colonies, horizontal transmission through the membrane feeding system seems to be the main source of virus propagation (Figure 2A). Quantitative PCR analyses revealed that asymptomatic and especially symptomatic flies release large amounts of virus into the blood at each meal. The high concentration of flies (75 flies per cage) feeding at the same time on a restricted blood volume and membrane surface and the successive feeding on the same membrane for economic reasons of up to ten cages of flies explains why, in laboratory colonies, 100% of adult flies were infected [33]. Furthermore, flies with SGH do not have to face the feeding difficulties found under field conditions, and even with a reduced life span, they represent an important source of virus contamination by releasing more virus into the blood diet during feeding, leading to a progressive increase of symptomatic flies in the colony.

## SGHV of Tsetse: Hope or Hindrance for Tsetse Control?

Many insect pathogenic viruses are currently used as biological control agents, e.g., baculoviruses in lepidopteran hosts [40], [41]. First investigations on SGHV led to the hypothesis that the virus could be related to the baculovirus group [20], which raised the possibility of its use as a biological control agent [42]. However, further studies contradicted this view [29], [30]. As mentioned above, in wild tsetse populations the virus is essentially transmitted vertically from mother to progeny and it is unlikely that horizontal transmission plays a significant role during feeding on animal hosts. Likewise, there is no evidence as yet for horizontal transmission of the virus through contact between flies, mating, or fecal contamination. These observations represent fundamental limitations to using the virus as a biological control agent, which requires an efficient way to deliver the virus to the target host.

From a purely practical point of view, the development of a biopesticide to control tsetse flies based on SGHV faces several technical challenges, a major one being the mass production of the virus for field application and its formulation. Artificial infection of healthy flies by feeding on contaminated blood is efficient, but the symptoms of SGH corresponding to highest virus loads are observed only in the next generation. Attempts to multiply the SGHV in an alternative host (with a short life cycle and easy to produce en masse, i.e., house flies) were not successful. Furthermore, no in vitro system to multiply the virus in cell culture is presently available. Finally, the formulation of a virus suspension allowing the virus particles to retain their infectivity under field conditions appears insuperable. Unlike baculoviruses, SGHV does not produce occlusion bodies and the virus envelope is extremely fragile. More than 90% of a purified virus suspension looses its infectivity after 3 days at 4°C (unpublished data). These difficulties make the use of this virus as a biological control agent impractical.

## Potential Virus Management Strategies

In view of our current understanding of the epidemiology of the virus under laboratory conditions, two possible strategies to manage the infection in a colony emerged: one based on the reduction or inhibition of horizontal virus transmission, the other on the reduction or inhibition of virus replication to avoid the development of symptomatic infections in a colony. To mitigate horizontal transmission in laboratory colonies, two approaches are being explored: (a) changing the feeding protocol currently used in colony rearing and/or (b) neutralizing the virus released during blood feeding by adding virus-specific antibodies to the blood. To reduce virus replication, two approaches have been tested: (c) inhibiting replication with commercially available antiviral drugs used to inhibit the DNA polymerase of similar viruses and (d) developing RNA interference to silence essential virus specific gene(s).

### Development of a Virus Management System Based on Modifying the Membrane Feeding Protocol

As mentioned above, the in vitro membrane feeding protocol using successive feeds on the same membrane favors horizontal virus transmission from infected to healthy colony flies. However, feeding the flies on fresh, unused (i.e., clean) blood and membrane at each meal (referred to as “clean feeding”) resulted in a four log reduction of the average virus load per fly (from 10^6^ to 10^2^ virus particles per fly) in three generations [35]. These results prompted the establishment of a clean feeding colony by feeding the flies and their progeny continuously on clean blood for more than 2 years. Monitoring of the virus load and of the prevalence of SGH in this colony showed a significant decrease of the virus load and an absence of SGH syndrome in dissected flies (unpublished data). Despite these very encouraging results, this approach is presently too costly to be applied in large-scale mass rearing, and a combination of clean feeding with other virus management strategies is required. One potential option is using clean feeding as a temporary filter to produce flies with low virus infection to be used in large-scale rearing in combination with other virus management methods [35].

### Neutralizing Virus Infection by Mixing Virus-Specific Antibodies with the Blood

Neutralizing antibodies are commonly used to control virus infections in vertebrates [43]–[49] and also to protect shrimps against a wispovirus [50]. Research on the use of neutralizing antibodies produced against GpSGHV structural polypeptides to potentially control horizontal transmission of the virus under in vitro membrane feeding has been initiated. To that end, a proteomic analysis of purified GpSGHV virus particles by liquid chromatography tandem mass spectrometry (LC-MS/MS) was undertaken. Sixty-one virion proteins were identified [51] and SGHV-specific antibodies against envelope dominant proteins were produced in rabbits by injecting purified recombinant proteins or synthetic oligopeptides. Neutralizing tests of SGHV infection using these antibodies are underway. After selecting the antibody(ies) capable of neutralizing SGHV infection, the corresponding protein(s) could be produced on large amounts using bacterial or baculovirus expression systems and used to produce antibodies in large animals. Appropriate concentrations of neutralizing antibodies could then be added to the blood meal. Combining the administration of antibodies with clean feeding or other virus management methods could be effective in keeping the virus infection at an acceptable level in tsetse colonies.

### Blocking SGHV Replication Using Commercial Antiviral Drugs

The discovery in the late 1970s that acyclic nucleoside analogs, in particular acyclovir, could inhibit DNA replication of herpes simplex virus (HSV) at concentrations far below those that affect cellular DNA synthesis sparked a new era in antiviral chemotherapy [52]. The underlying reason for the selectivity—that acyclovir is specifically converted to the active metabolite by an HSV-encoded thymidine kinase— was unexpected, but the potential for exploiting viral enzymes to develop potent and specific antiviral drugs was clearly demonstrated. Acyclovir subsequently became a successful treatment for HSV-1 and HSV-2 [52], [53]. After completing the genome sequence of GpSGHV, the phylogenetic analysis of its DNA polymerase amino acid sequence unexpectedly revealed that it shares high similarity with herpes virus DNA polymerase [29]. These similarities led us to speculate that acyclovir might have an antiviral effect on GpSGHV replication. Preliminary results indicated that *per os* treatment of flies by adding acyclovir and valacyclovir to the blood meals significantly reduces viral loads (unpublished data). Several other antiviral drugs are currently available for DNA viruses that could be screened to assess their impact on GpSGHV DNA replication. Several parameters, such as (i) absence of any negative effect on fly survival and productivity, (ii) significant reduction of virus loads, (iii) suitable bioavailability when offered to the flies mixed with blood, and (iv) affordable price will have to be considered for selecting appropriate antiviral drugs for use in large-scale tsetse rearing facilities. Administration of one or more antiviral drugs could be combined with other methods of virus management.

### Inhibiting SGHV Infection by Silencing Virus-Specific Genes Using RNAi Technology

RNA interference (RNAi) has recently emerged as a powerful tool for specific gene silencing in gene therapy [54], [55]. Recent studies have reported the successful use of dsRNAs produced in a bacterial system as therapeutic agents for economically affordable oral treatment of white spot syndrome disease of shrimps [56], [57]. The potential of a similar approach based on the silencing of essential GpSGHV gene(s) such as DNA polymerase, p74, or *per os* infectivity factors to mitigate the SGHV infection is worth evaluating.

In conclusion, the GpSGHV represents a threat to integrated control programs against *G. pallidipes* that incorporate the release of sterile males because it reduces the productivity of *G. pallidipes* colonies, which in certain circumstance has resulted in the collapse of the colony. The virus cannot be used as a biological control agent due to several limitations. So far the virus has been studied from the point of view of its impact on *G. pallidipes* colonies in a rearing facility, but the impact of the virus on release programs in terms of competiveness, performance, and mating behavior of symptomatic and asymptomatic infected male flies still requires further study.

**Key Learning Points**

- Tsetse salivary gland hypertrophy syndrome was reported in wild tsetse populations from several species in different countries with a prevalence of 0.5%–15%, while in *G. pallidipes* laboratory colonies prevalence can reach 85%.
- Tsetse salivary gland hypertrophy virus (SGHV) cannot be used as a biological control agent in tsetse control program due to the virus characteristics, i.e., fragile structure of virus particles and limited role of horizontal virus transmission.
- GpSGHV seriously impedes rearing of *G. pallidipes* in large-scale facilities by reducing fly productivity, which hinders SIT programs for tsetse control.
- A virus management strategy based on reducing horizontal virus transmission by changing the blood feeding system currently used, neutralizing virus infection using virus-specific antibodies, and reducing virus replication by administration of antiviral drugs or RNAi technology is being developed.

**Five Key Papers in the Field**

- Whitnall ABM (1934) The Trypanosome infections of *Glossina pallidipes* in the Umfolosi Game Reserve, Zululand. Onderstepoort J Vet Sci Anim Ind 2: 7–21.
- Jaenson TGT (1978) Virus-like rods associated with salivary gland hyperplasia in tsetse, *Glossina pallidipes*. Trans R Soc Trop Med Hyg 72: 234–238.
- Jura WGZO, Odhiambo TR, Otieno LH, Tabu NO (1988) Gonadal lesions in virus-infected male and female tsetse, *Glossina pallidipes* (Diptera: Glossinidae). J Invertebr Pathol 52: 1–8.
- Abd-Alla AMM, Cousserans F, Parker AG, Jehle JA, Parker NJ, et al. (2008) Genome analysis of a *Glossina pallidipes* salivary gland hypertrophy virus (GpSGHV) reveals a novel large double-stranded circular DNA virus. J Virol 82: 4595–4611. doi:10.1128/JVI.02588-07.
- Abd-Alla AMM, Kariithi H, Parker AG, Robinson AS, Kiflom M, et al. (2010) Dynamics of the salivary gland hypertrophy virus in laboratory colonies of *Glossina pallidipes* (Diptera: Glossinidae). Virus Res 150: 103–110.
